# In-house 3D printed porous implants: does strontium-coating enhance osseointegration? An *in-vivo* study in large animals

**DOI:** 10.3389/fsurg.2026.1818358

**Published:** 2026-05-22

**Authors:** Anna B. Borgognoni, Sarah S. Freund, Jørgen Baas, Michael M. Bendtsen, Jeppe S. Byskov, Bahram Ranjkesh, Jens R. Nyengaard, Ruben Pauwels, Klaus P. Almtoft, Thomas Baad-Hansen

**Affiliations:** 1 Department of Orthopaedic Oncology, Aarhus University Hospital, Aarhus, Denmark; 2 Additive Manufacturing, Danish Technological Institute, Aarhus, Denmark; 3 Department of Dentistry and Oral Health, Aarhus University, Aarhus, Denmark; 4 Core Center for Molecular Morphology, Section for Stereology and Microscopy, Department of Clinical Medicine, Aarhus University, Aarhus, Denmark; 5 Department of Pathology, Aarhus University Hospital, Aarhus, Denmark; 6 Tribology Center, Danish Technological Institute, Aarhus, Denmark

**Keywords:** 3D printing, histomorphometry, osseointegration, porous surface design, push-out test, strontium, titanium implants

## Abstract

**Introduction:**

Early osseointegration is essential for the long-term success of uncemented custom-made prostheses, particularly in oncologic bone reconstruction where bone stock is compromised. Strontium (Sr) has demonstrated osteogenic potential, and Ti-Sr-O surface coatings may enhance local bone formation.

**Methods:**

This study evaluated whether Ti-Sr-O coating improves early osseointegration of 3D-printed porous Ti6Al4 V implants in a large-animal 2 mm gap model. Twenty female Romney-Texel sheep were randomized to receive either uncoated (P1) or Ti-Sr-O–coated (Sr-P1) cylindrical porous implants in the proximal tibia (*n* = 10/group). After four weeks, implant fixation was assessed using axial push-out testing, and blinded histomorphometric analysis quantified bone, fibrous tissue, and membrane fractions in defined regions of interest. Biomechanical outcomes included ultimate shear strength, apparent shear stiffness, and energy absorption. Data were analyzed using the Mann–Whitney U test (*α* = 0.05).

**Results:**

All animals completed follow-up without implant-related complications. No significant differences were observed between groups in shear strength (*p* = 0.85), stiffness (*p* = 0.91), or energy absorption (*p* = 1.00). Histomorphometry demonstrated comparable bone volume fractions and bone–implant contact between groups (*p* > 0.05), with both groups exhibiting early woven bone formation and no foreign body reaction.

**Discussion:**

Within the limitations of this gap model and four-week observation period, Ti-Sr-O coating did not enhance early mechanical fixation or osseointegration, highlighting that, within the constraints of this gap model and observation period, the study may be insufficient to detect the localized effects of Sr release, and that alternative experimental designs should be considered.

## Introduction

In patients diagnosed with bone sarcoma, the bone stock can be compromised either due to neoadjuvant therapies or due to the extensive surgical resection (1). Under these challenging conditions, the design of an uncemented custom-made prosthesis becomes particularly important and long-term success depends heavily on achieving early osseointegration. Therefore, surface modification has become a central research focus (2). Among the various bioactive coatings explored, hydroxyapatite (HA) is the most established (3), yet interest is increasing in alternatives that may further enhance bone–implant integration. Strontium (Sr) is an alkaline metal that, because of its similarity to calcium, can be incorporated into the mineral phase of bone (4). It has been shown that Sr has a positive effect on peri-implant bone healing if administered systemically (5). However, these interventions carry a substantial risk of adverse events like headache, nausea, and diarrhea; therefore, interest in the localized delivery of Sr at the bone/implant interface has increased. A promising development in this field is a Ti-Sr-O coating introduced by Andersen et al. (6). This surface coating provides sustained Sr release and demonstrates high mechanical stability. In a rat model, Ti-Sr-O–coated implants significantly accelerated bone ingrowth compared with uncoated controls (6). These findings highlight the potential of Sr-based coatings to enhance the performance of 3D-printed porous implants by improving early and long-term fixation.

Three-dimensional printing (3DP) has transformed the development of orthopaedic implants by enabling the rapid creation of patient-specific devices with complex geometries. For patients with bone sarcomas, 3DP allows surgeons to design prostheses tailored to the large and irregular defects created by tumour resections (7, 8). These challenges have driven the establishment of in-house hospital 3DP centres (9), such as the one at our institution, which collaborates with the Danish Technological Institute (DTI) for metal 3DP manufacturing. Previous works (10, 11) have led to the development of porous implant designs and a validated gap model for assessing osseointegration (12–15).

The present study aimed to evaluate the effect of Ti-Sr-O coating on early osseointegration in a large-animal model by comparing coated implants with uncoated controls. The null hypothesis is that coated implants will present the same osseointegration compared to non-coated implants.

## Materials and methods

### Study design

The study was designed as an unpaired comparison within two groups. Each animal received one implant in the left proximal tibia. ISO guidelines recommend an observation time of 1–4 weeks’ for assessing short-term outcomes (10). The observation time was therefore set at 4 weeks. Specimens were then collected and cut into blocks with the implant and surrounding tissue; the outermost 1 mm end disc was discarded. Each block was further divided into a 3 mm segment for mechanical testing and a 6 mm segment for histomorphometric analysis.

### Animals

Twenty female Romney-Texel sheep with a mean weight of 65.5 Kg (58–75.6) and a mean age of 41.5 months (36–50) were included in the study. The study was approved by the Danish Animal Research Inspectorate (ID: 2022-15-0201-01240), conformed to Danish law and the ARRIVE guidelines (16) were followed. Sheep as a large animal model were chosen in accordance to a recent systematic review from Spece et al. (3), where sheep are shown to be the most used model for large size animal. Adult sheep has similarities in weight, metabolism, and bone remodelling rates to humans (10). Furthermore sheep´s metaphysis cancellous bones are ideal to accommodate experimental implants that can mimic clinical implant surface technologies (10). The sheep had no prior experimental exposure. After a one-week acclimatization, they roamed freely outdoors during observation. Pre- and post-operative care was provided daily by trained staff under veterinary supervision. For the first three days after surgery, the sheep were checked three times daily.

### Sample size

A sample size calculation for unpaired randomized studies was done. The assumptions for the sample size estimations were based on effect sizes reported in previous studies using similar gap models but different surface modifications (13). The power was set at 80% and the significance level at 5%. Based on the sample size estimations and from previous studies with a similar model (12–14, 17) it was decided to include twenty sheep, for a total of 2 sets of 10 implants.

### Implant design and production

**Figure 1:**
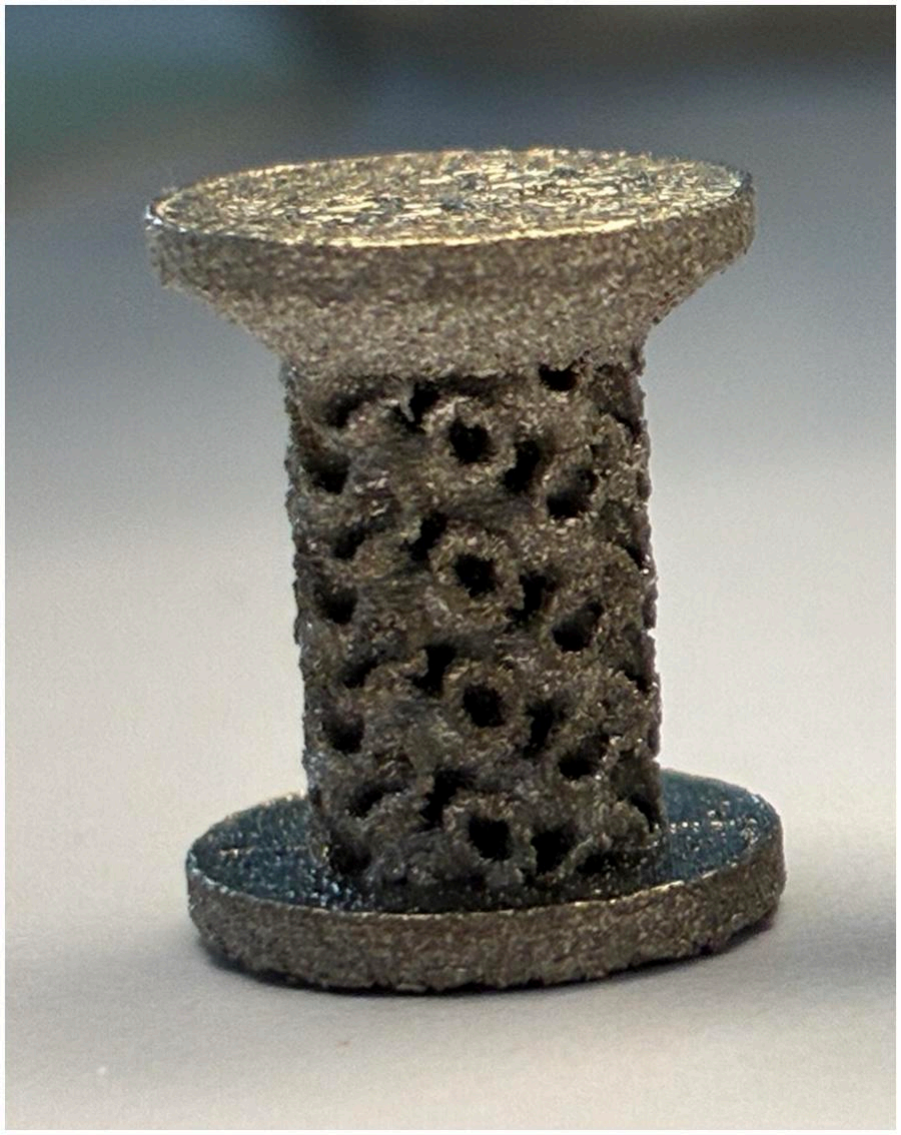
Gap-models utilized.

Both implants (Figure 1) exhibited mean pore sizes (P1: 630 µm) and porosities (P1: 73.85%) consistent with values reported in current literature. Ti-Sr-O-coating was applied to one group.

### Implant design, fabrication and coating

Implant were manufactured at the Danish Technological Institute, Aarhus, Denmark, using a selective laser manufacturing machine (SLM® Twin, SLM Solutions, Lübeck, Germany) with a twin 400W IPG fibre laser with a layer thickness of 60 µm. The Ti6Al4V-grade 23 ELI powder (SLM Solutions, Lübeck, Germany) used had a particle size between 20 and 63 µm and a mass density of approximately 4.43 g/cm^3^. All implants were cylindrical, 10 mm tall and 6 mm in diameter, each with a 10 mm diameter, 1 mm high footplate at both ends. In a 10 mm drill hole, they were centred and left a uniform 2 mm gap around the implant. The post processing consisted in ultrasound cleaning with ethanol and water to eliminate the remaining powdeŕs particles.

The coating process was developed at DTI and applied using direct-current magnetron sputtering with an industrial PVD coating system (CemeCon AG, Germany). This plasma-based technique coats implants by transferring material from a target onto the surface to be coated. In this process, the plasma used for deposition is based on argon gas. Ion bombardment of the target material initiates a collision cascade, which results in the emission of atoms that then condense on the implant's surface (18, 19).

The methods used in this study followed protocols previously detailed by Andersen et al. (6). Test implants were mounted on a rotating device inside a vacuum chamber with a base pressure of 1 mPa. Argon at a pressure about 1.1 Pa served as the sputtering gas. The targets used for the depositions were a Grade 1 Ti target with a purity of 99.5% and a sintered composite target made from 50% SrTiO3% and 50% Ti (w/w) mixed as powders with all components having a purity of 99.9%. Using this the coating consisted of a 100 nm adhesion layer followed by a 1-µm Ti-Sr-O layer.

### Sterilization

Sr-coated implants pose the unique problem that the coating is vulnerable to water, humidity, and heat, as by design, the releasing of Sr instantly starts with water contact. Consequently, an alternative and equivalent sterilization method called e-beam (rapid, high-dosage radiation method that uses high-energy electrons to disrupt the DNA of microorganisms, rendering them sterile), described in other studies (20–22), was used in this study. Implants were sterilized at Sterigenics Denmark A/S, with a dose of 25 kGy.

### Anaesthesia, pain management and handling of animals

Sheep were given intramuscular Noromox (150 mg/mL) at a dose of 15 mg/kg both before and after surgery to prevent infection. Metacam was used for pain relief during the same periods. After surgery, a fentanyl transdermal patch (75 µg/h) was applied for three days to manage postoperative pain. All animals were allowed to bear weight without restriction.

### Surgery

**Figure 2:**
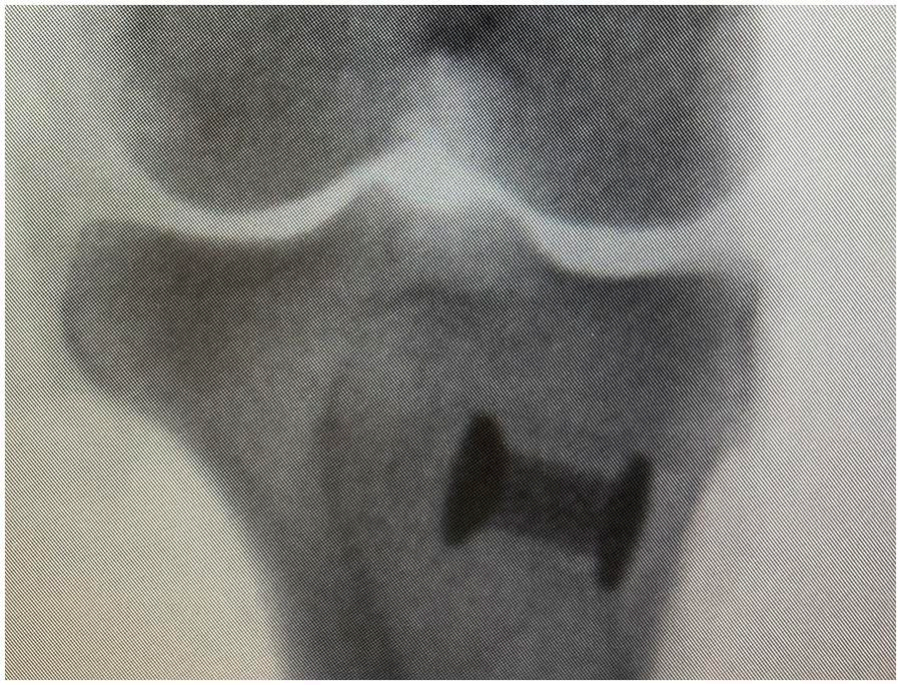
Intraoperative x-ray image.

Under general anaesthesia and sterile conditions, a 5 cm incision was made over the proximal tibia with electrocautery. A guide wire was inserted perpendicularly to the tibial plateau distal to the epiphysis and confirmed by intraoperative x-ray. A 12 mm deep, 10 mm wide cavity was drilled, excess periosteum and bone fragments were removed, and implants were centrally placed with an impaction tool (Figure 2). All implants were inserted by the same experienced surgeon. After four weeks, sheep were sedated and euthanized with an overdose of barbiturates.

### Specimen preparation

**Figure 3:**
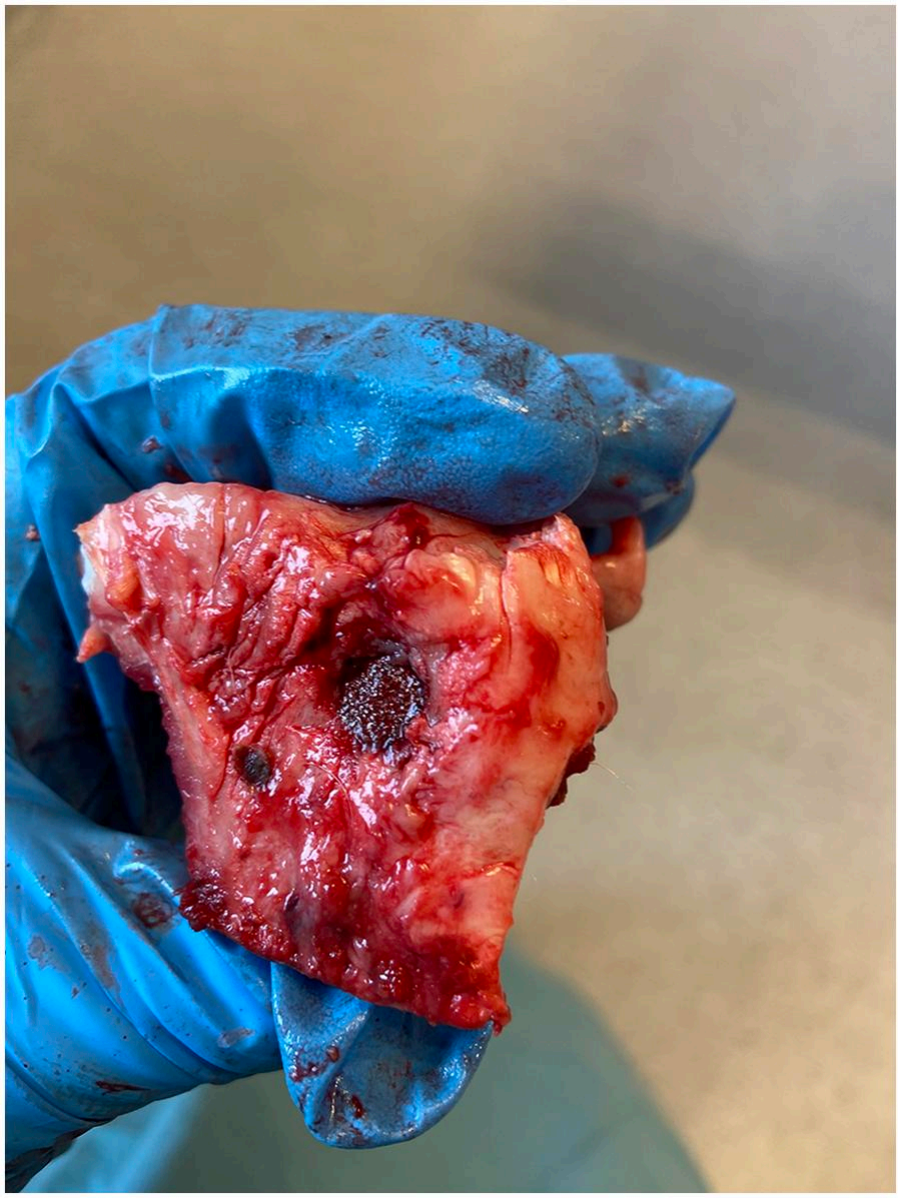
Sample at retrieval.

The proximal tibia (Figure 3) was cut perpendicular to the implant axis using a water-cooled diamond band saw (Exact Apparatebau, Nordenstedt, Germany). A 3 mm outer section was stored at −20 °C for mechanical tests, while the inner section was dehydrated in graded ethanol (70%–100%), embedded in methylmethacrylate (Technovit 7200 VCL; Exact Apparatbau, Nordenstedt, Germany), and sliced into four 50 μm vertical central sections with a microtome (KDG-95, MeProTech, Heerhugowaard, The Netherlands). These were stained with toluidine blue, mounted on slides, and analysed to distinguish woven from lamellar bone by morphology.

### Biomechanical testing using push-out test

**Figure 4:**
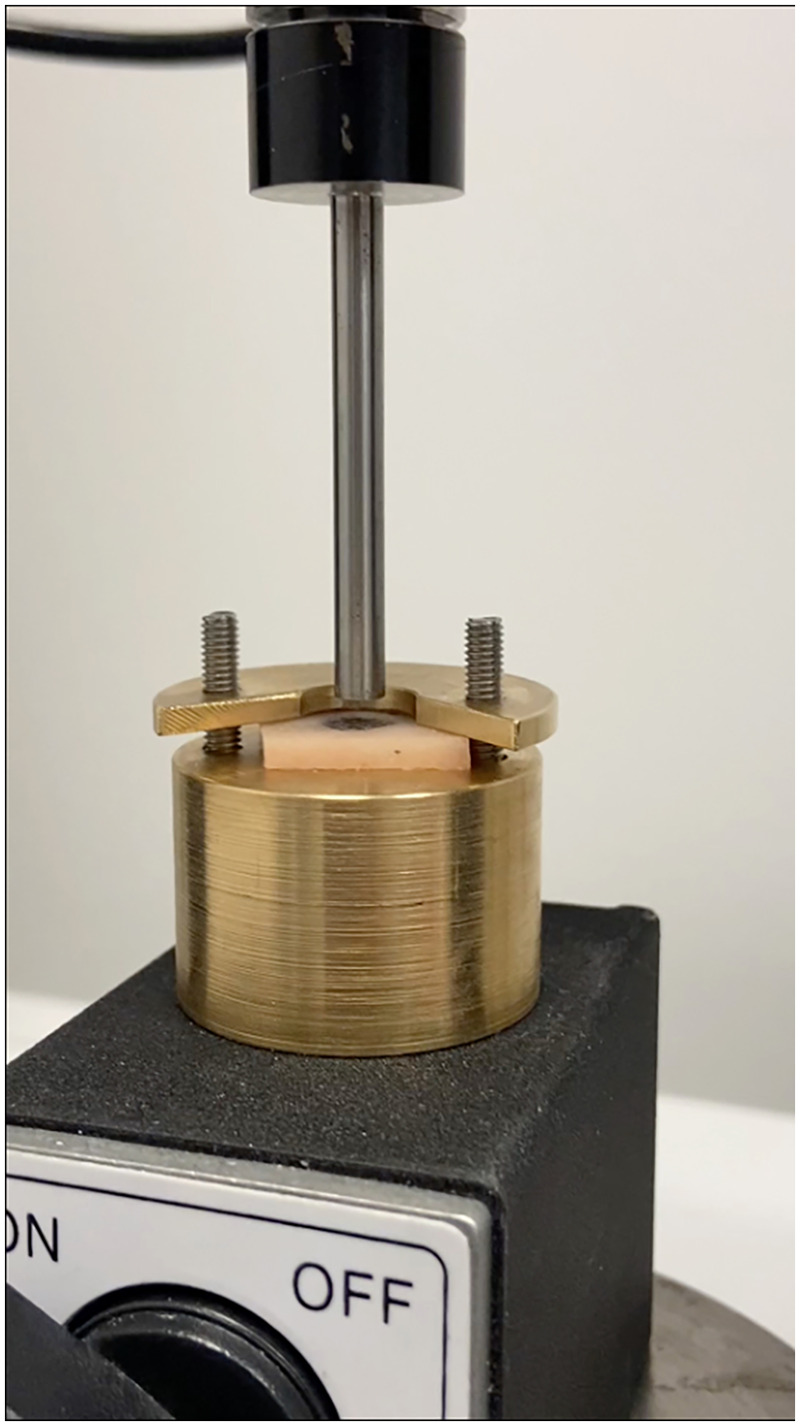
Mechanical tests using push-out test.

Each embedded sample, comprising an implant encased in bone, was mounted in a custom-designed fixture featuring a 7.4-mm central aperture. The specimens were oriented with the cortical surface facing upward. Axial push-out tests were then performed using a universal testing machine (Instron Universal Testing Machine; Instron Ltd., High Wycombe, UK) equipped with a 5-mm-diameter metallic loading pin. During testing, the specimens rested on a support jig with a 7.4-mm opening, and the implant was displaced at a crosshead speed of 5 mm/min following the application of a 0.5 N preload (Figure 4). Length and diameter of each implant were measured with a micrometre after each test, and the surface area was calculated. Load (N) and displacement (mm) data were used to determine three parameters, described by Søballe (23): ultimate shear strength (the maximum load the connection can withstand normalized by an approximation of the surface area), apparent shear stiffness (a measure of a shear connector's resistance to deformation, calculated from the force-slip or force-displacement data recorded during the test), and total energy absorption (how much energy a material or composite can absorb when a compressive force is applied to push it out or deform it).

### Histomorphometry

**Figure 5:**
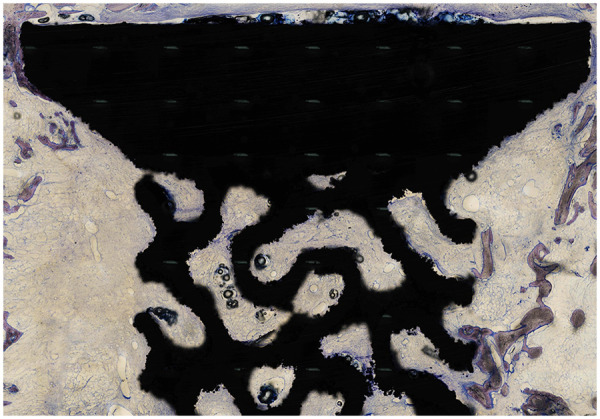
Histomorphological slide.

Four central sections, each 50 µm thick, were sliced parallel to the implant axis using the Vertical Uniform Random (VUR) sectioning technique on a hard-tissue microtome (Figure 5). Two regions of interest (ROIs) were established in Fiji (24), an open-access image processing software, with the help of a semi-automated macro script. Zone 1 (surface zone) was defined from −500 μm extending into the implant and +500 μm into the 2 mm gap. Zone 2 (gap zone) continues from the end of Zone 1, spanning between 500 and 1500 μm into the gap.

Blinded quantitative histomorphometry was conducted using both Fiji and new CAST (Visiopharm A/S, Hoersholm, Denmark). To measure relative volumes according to stereological principles, a systematic set of test points was employed. In Zone 2, a 100% sampling fraction was employed, ensuring comprehensive observation of the entire region. Volume fractions of new bone, fibrous tissue, and foreign body membranes were quantified, with an average of 827 points assessed per slide within this zone. For Zone 1, direct evaluation of bone growth on the implant surface was conducted using a sine-weighted line-intersection technique, which was deemed most appropriate for this measurement. On average, 232 line-intersections per slide were recorded.

### Statistical analysis

All statistical analyses were made using R (v4.3.1; R Core Team 2021). The datasets from mechanical and histological tests were tested for normality with the Shapiro–Wilk test. The data were not normally distributed and were evaluated with the Mann–Whitney U test. For all comparisons, the significance level was set at 0.05.

A sensitivity (detectable effect size) analysis was performed This analysis indicates that, with *n* = 10 per group and *α* = 0.05, the study was adequately powered (80%) to detect moderate-to-large effect sizes (approximately standardized effect size d ≈ 0.8–0.9), but not small differences.

## Results

All 20 sheep resumed full weight bearing within two days post-surgery and completed the four-week observation period without exhibiting distress or other procedure-related complications. Only one animal displayed signs of superficial infection, which was managed successfully with antibiotics. At harvest, no implants exhibited macroscopic and microscopic evidence of infection.

### Biomechanical testing

Axial push-out testing showed no significant differences between uncoated (P1) and Ti-Sr-O–coated (Sr-P1) implants across all parameters (Table 1).

**Table 1:** P1 vs. Sr-P1, Biomechanical results. Results are reported as [median (IQR)].

Implant		Biomechanical characteristics	
	Strength (MPa)	Energy (J/m^2^)	Stiffness (MPa/mm)
P1	0.08 (0.10)	31.00 (20.60)	0.30 (0.30)
Sr-P1	0.08 (0.17)	28.50 (36.90)	0.27 (0.54)
Mann–Whitney U test	*p* = 0.85	*p* = 1	*p* = 0.91

Median ultimate shear strength was identical in both groups (0.08 MPa; *p* = 0.85). Apparent shear stiffness was similar between P1 (0.30 MPa/mm) and Sr-P1 (0.27 MPa/mm; *p* = 0.91). Energy absorption also did not differ, with median values of 31.00 J/m^2^ for P1 and 28.50 J/m^2^ for Sr-P1 (*p* = 1.00).

### Histomorphometry

#### Zone 2 (500–1500 µm)

No significant differences in tissue composition were observed between groups (Table 2). Bone volume fraction was identical (median 0.09; *p* = 0.45), while fibrous tissue predominated in both groups (0.82 vs. 0.74; *p* = 0.21). No membrane formation was detected.

**Table 2:** P1 vs. Sr-P1, Histomorphometric ingrowth in zone 2. Results are reported as [median (IQR)].

Implant		Tissue volume fractions	
	Bone	Membrane	Fibrous
P1	0.09 (0.05)	0	0.82 (0.14)
Sr-P1	0.09 (0.13)	0	0.74 (0.18)
Mann–Whitney U test	*p* = 0.45	-	*p* = 0.21

#### Zone 1 (−500 to +500 µm)

Bone–implant contact was minimal and comparable between groups (0.00 vs. 0.01; *p* = 0.53) (Table 3). Fibrous tissue dominated the interface in both groups (1.50 vs. 1.50 µm⁻^1^; *p* = 0.60), with no membrane formation observed.

**Table 3:** P1 vs. Sr-P1, Implant surface ongrowth in zone 1. Results are reported as [median (IQR)].

Implant	Surface area fractions (Sv, μm^−1^)		
	Bone	Membrane	Fibrous
P1	0.00 (0.02)	0	1.50 (0.27)
Sr-P1	0.01 (0.03)	0	1.50 (0.14)
Mann–Whitney U test	*p* = 0.53	-	*p* = 0.60

#### Qualitative observations

Both groups exhibited early woven bone formation without lamellar organization. The implant surface was largely separated from bone by fibrous tissue. No inflammatory or foreign body reactions were identified.

## Discussion

In the present large animal study, we hypothesised that TI-Sr-O-coating could enhance the osseointegration of in-house 3DP porous Ti-6Al-4 V implants in a 2 mm gap model. However, no significant improvement in early mechanical strength and implant stability could be demonstrated for the coated group. A previous study in a rabbit model from Christensen et al. (18) has shown that Sr from the implant surface only diffuses a few hundred micrometres into the bone. In the current study, the osteogenic effect of Sr is also likely concentrated in the immediate vicinity of the implant surface were the direct bone-contact is scarce, and Zone 2 is presumably located outside Sr's biological radius of action. The model design may therefore be inherently insensitive to detecting the intended coating effect on bone ingrowth and results should therefore not be interpreted as biological inactivity of the coating, but rather as insufficient local exposure and consideration should be given to press-fit or semi–press-fit, a smaller gap, longer follow-up, or alternative Sr release profiles.

A previous study from Vestermark et al. (12) have combined Sr coating with HA, suggesting that due to the low solubility of the HA layer, Sr incorporated within the HA coating may not effectively reach the interface. Previous studies using the Ti-Sr-O coating reported that the peak release occurred within the first 24 h after implantation (6). In the current study the Sr coating was deposited directly on the surface and was evenly distributed on the surface, without sealing the implants pores (coating thickness is only around 1 μm and does not influence the pore size). At microscopical evaluation, the two groups of implants seemed indistinguishable, allowing the blinding of the process, but no further analysis was conducted to evaluate the eventual presence of residual coating on the implant.

Axial push-out testing primarily assesses macroscopic mechanical fixation rather than microscopic alterations in the bone–implant interface. Accordingly, histological changes in bone quality or bone–implant contact may occur without translating into detectable differences in mechanical performance. Osseointegration is initiated by any injury to the pre-existing bone matrix. When the matrix is exposed to extracellular fluid, non-collagenous proteins and growth factors are released, triggering bone repair. Once initiated, osseointegration proceeds according to a common, biologically regulated sequence comprising three stages: incorporation through woven bone formation; adaptation of bone mass to functional loading via lamellar and parallel-fibered bone deposition; and adaptation of bone structure to load through bone remodelling (25). Consequently, although push-out tests are well established in gap models, they might be insensitive to early modifications at the bone-implant interphase and may be more appropriate for detecting differences between implants at later stages of osseointegration.

While a press-fit model would likely have demonstrated a greater mechanical effect of the coating, such a model was deliberately avoided because the reduced gap would not adequately represent the clinical oncological setting and would therefore limit the relevance of the findings in this context. Additionally, implant fixation may result from either bone bonding or mechanical interlocking (26). The gap model was designed specifically to address bone bonding alone.

Histomorphological evaluation demonstrated that both implant groups were in the early phase of woven bone formation. Although ISO guidelines recommend an upper observation limit of 4 weeks for assessing early osseointegration in large animal models, the specific experimental model should be taken into account. In the case of a 2-mm gap model, the observation period may need to be adjusted, or the evaluation limited to confirming implant tolerance and assessing the presence or absence of a foreign body reaction. All the implants in this study were well tolerated, with no microscopical signs of foreign body reaction. Comparisons between studies utilizing similar animal models are challenging due to substantial variations in study design. However, recent research (27, 28) indicates that late-stage osseointegration analysis may more effectively demonstrate the advantages of 3D-printed porous implants. Longer observation periods (e.g., 8 weeks or beyond) may reveal different outcomes, particularly regarding bone maturation and long-term implant stability.

Generally, it was observed that the model design and implant production were accomplished quickly and efficiently with a lead-time of approximately one week, from implant design to production.

### Limitations

The valid sample size calculation was based on prior studies; however, it is important to acknowledge that these studies utilized different coatings, and the effects of the current Sr-coating in a 2 mm gap model remain unknown. Under these premises, in this study the coating did not appear to either enhance or diminish the mechanical or histomorphometric parameters.

The 4-week time point was selected to specifically evaluate early-stage osseointegration, following the recommendation in the ISO guidelines, but we acknowledge that longer observation periods (e.g., 8 weeks or beyond) may reveal different outcomes, particularly regarding bone maturation and long-term implant stability.

No post-implantation surface analysis (e.g., XRF or EDS) was performed in this study, which represents a limitation. The coating method applied follows previously validated protocols demonstrating strong adhesion and controlled Sr release, as described by Andersen et al. (6) However, the absence of coating verification (either pre-implantation controls or post-retrieval analysis), make it impossible to determine whether the observed lack of effect reflects biological inactivity of the coating or potential degradation/loss under the applied condition. Future studies should include surface characterization of implants after sterilization and prior to implantation, retrieval analysis to confirm coating persistence and quantification of local Sr release.

A sample of *n* = 10 per group was based on prior studies using different coatings, due to the unknown effect of Ti-Sr-O coating in a 2 mm gap model. The study was powered to detect moderate-to-large differences comparable to prior coating studies; however, it may be underpowered to detect small or spatially localized biological effects, particularly given the limited diffusion radius of Sr in this model.

## Conclusion

As per our aims, we investigated whether SrTi-O-coating could enhance osseointegration and mechanical stability of porous titanium alloy implants in 2 mm gap model. Within the limitations of this 2 mm gap model and short observation period, no differences were detected between coated and uncoated implants. However, given the localized biological action of strontium and the absence of coating verification under the present experimental conditions, these findings should not be interpreted as evidence of coating inefficacy. Rather, they underscore the importance of model selection and verification strategies when evaluating surface modifications in translational settings.

## Data Availability

The raw data supporting the conclusions of this article will be made available by the authors, without undue reservation.
